# Prokineticin 2 (PK2) Rescues Cardiomyocytes from High Glucose/High Palmitic Acid-Induced Damage by Regulating the AKT/GSK3*β* Pathway In Vitro

**DOI:** 10.1155/2020/3163629

**Published:** 2020-05-18

**Authors:** Zhen Yang, Yin Wu, Linge Wang, Peng Qiu, Wenliang Zha, Wei Yu

**Affiliations:** ^1^Department of Pharmacology, School of Pharmacy, Hubei University of Science and Technology, Xianning, Hubei 437100, China; ^2^Department of Surgery, Clinic Medical College, Hubei University of Science and Technology, Xianning, Hubei 437100, China; ^3^National Demonstration Center for Experimental General Medicine Education, Hubei University of Science and Technology, Xianning, Hubei 437100, China

## Abstract

Prokineticin 2 (PK2) is a small 8 kDa protein that participates in many physiological processes, such as angiogenesis, inflammation, and neurogenesis. This experiment investigated the effect of PK2 on high glucose/high palmitic acid-induced oxidative stress, apoptosis, and autophagy in cardiomyocytes and the AKT/GSK3*β* signalling pathway. H9c2 cells were exposed to normal and high concentrations (33 mM) of glucose and palmitic acid (150 *μ*M) with or without PK2 (10 nM) for 48 h. Reactive oxygen species were detected using the fluorescent probes DCFH-DA and DHE. Changes in apoptosis were assessed using flow cytometry, and autophagosomes were detected using Ad-GFP-LC3. Apoptotic proteins, such as Cleaved Caspase3, Bax, and Bcl-2; autophagy proteins, including Beclin-1 and LC3B; and PK2/PKR/AKT/GSK3*β* signals were evaluated using western blotting. Cardiomyocytes exposed to high glucose/high palmitic acid exhibited increases in intracellular ROS, apoptosis, and autophagosomes, and these increases were robustly prevented by PK2. In addition, high glucose/high palmitic acid remarkably suppressed PK2, PKR1, and PKR2 expression and p-AKT/AKT and p-GSK3*β*/GSK3*β* ratios, and these effects were significantly prevented by PK2. Moreover, an AKT1/2 kinase inhibitor (AKT inhibitor, 10 *μ*M) blocked the effects of PK2 on the changes in cardiomyocyte exposure to high glucose/high palmitic acid. These results suggest that PK2 attenuates high glucose/high palmitic acid-induced cardiomyocyte apoptosis by inhibiting oxidative stress and autophagosome accumulation and that this protective effect is most likely mediated by the AKT-related signalling pathway.

## 1. Introduction

Diabetes is a metabolic disease characterized by hyperglycaemia and is becoming a global health problem [1]. The International Diabetes Federation predicts that the total number of diabetes cases will reach 700 million by 2045 [2]. Type 2 diabetes, which is associated with the disturbed metabolism of glucose and lipids, accounts for 90% of all the cases. Diabetes mainly harms the body's macro- and microcoronary arteries and poses a high risk for cardiovascular morbidity and mortality [3]. Diabetic cardiomyopathy (DCM) is a structural and functional disorder of the heart caused by diabetes that is independent of hypertension, coronary atherosclerotic heart disease, valvular heart disease, and other known heart diseases [4]. However, the mechanisms underlying DCM remain unclear.

Prokineticin 2 (PK2), a secreted 8 kDa protein [5], is involved in a variety of physiological and pathological processes, including nerve growth, immune response, angiogenesis, and inflammation [6–9]. PK2 binds to two receptors, namely, prokineticin receptor 1 (PKR1) and prokineticin receptor 2 (PKR2), which share approximately 85% amino acid identity, which are widely distributed in both mice and humans, and which modulate biological processes, such as neuronal survival and testis development [10–12]. In 2007, Urayama et al. first discovered that PK2 is expressed in cardiac tissues, including in H9c2 cardiomyocytes and H5V vascular endothelial cells [13]. PK2 not only promotes angiogenesis in H5V vascular endothelial cells but also inhibits H_2_O_2_-induced H9c2 injury [13]. However, the role, if any, of PK2 and PKR in pathological DCM remains unknown, and investigation of the role of PK2/PKR in type 2 diabetes-induced damage to cardiomyocytes is required.

AKT plays a crucial role in cardiac growth, coronary angiogenesis, metabolic regulation, and tumours [13–17] and mediates biological activities by inactivating its downstream target GSK3*β* [18]. A previous study showed that PK2 participates in neuroprotection by stimulating the ERK and AKT survival signalling pathways [9]. In addition, Su et al. demonstrated that PK2 relieves hypoxia/reoxygenation-induced injury in H9c2 cardiomyocytes by activating the AKT pathway [19]. However, whether PK2 contributes to cardiomyocyte survival or repairs high glucose/high palmitic acid-induced injury by activating the AKT/GSK3*β* signalling pathway is still unclear. Our current work reveals that PK2 is a potentially useful target for treating high glucose/high palmitic acid-induced cardiomyocyte damage. Herein, our subsequent work illuminates that PK2 is a potentially useful target for treating high glucose/high palmitic acid-induced cardiomyocyte damage.

## 2. Materials and Methods

### 2.1. Cell Culture and Treatment

The rat cardiomyocyte cell line H9c2 was purchased from the China Center for Type Culture Collection (CCTCC, China) and cultured in DMEM (HyClone, USA) containing 10% FBS (Gibco, USA) and 1% penicillin-streptomycin at 37°C in a humidified atmosphere (5% CO_2_ and 95% air). The cells were randomly divided into the following experimental groups: the normal (NG) group, which was exposed to 5.5 mM D-glucose, and the high glucose/high palmitic acid (HG-PA) groups, which were exposed to 33 mM D-glucose plus 150 *μ*M palmitic acid (Sigma, USA) for 48 h in the absence or presence of PK2 (Sigma, 10 nM) or AKT1/2 kinase inhibitor (AKT inhibitor, Sigma, 10 *μ*M) [20].

### 2.2. Analysis of Biochemical Parameters

Cardiomyocytes were treated with reagents for 48 h, and then physiological saline was added. After that, the cardiomyocytes were placed in a freezer at -20°C and repeatedly frozen/thawed three times. The suspension was centrifuged for 15 min at 4000 r/min, and the supernatant was homogenized to measure superoxide dismutase (SOD) and malondialdehyde (MDA) levels. The procedure strictly followed the instructions of the SOD assay kit and MDA assay kit (Nanjing Jiancheng Bioengineering Research Institute, China).

### 2.3. Flow Cytometry

Apoptosis was detected by an Annexin V-FITC/PI Apoptosis Detection Kit (Meilunbio, China). After being treated as described above, H9c2 cells were digested with trypsin, resuspended in a 1x binding buffer, and then incubated with Annexin V-FITC and PI for 15 min in the dark [21]. Flow cytometry was used to determine the effect of PK2 on the apoptosis rate of injured cardiomyocytes.

### 2.4. MTT Assay

MTT (3-(4,5-dimethylthiazol-2-yl)-2,5-diphenyltetrazolium bromide) was used to evaluate cell viability. H9c2 cells were seeded in 96-well plates and exposed to high glucose/high palmitic acid with or without PK2 or AKT inhibitor for 48 h. Then, 20 *μ*L of MTT working solution was added and incubated for 4-6 h. The MTT solution was removed, and 150 *μ*L of DMSO (Sigma) was added to each well. The absorbance of each well at 490 nm was measured.

### 2.5. Intracellular ROS Measurement

A Reactive Oxygen Species Assay Kit (Beyotime Biotechnology, China) was used to detect intracellular reactive oxygen species. Cardiomyocytes were incubated with the fluorescent probe dichlorodihydrofluorescein diacetate (DCFH-DA, 10 *μ*M) for 30 min. A Tecan fluorometric microplate reader was used to detect ROS products at 530 nm.

The fluorescence probe dihydrogen ingot (DHE) was used to measure intracellular superoxide anion levels. Cultured cardiomyocytes were incubated with 10 *μ*M DHE (Beyotime Biotechnology) and then cultured with 5 *μ*g/mL DAPI (Beyotime Biotechnology) for 10 min. H9c2 cardiomyocytes were observed by fluorescence microscopy.

### 2.6. Ad-GFP-LC3 Transfection

An adenovirus-expressing GFP-LC3 fusion protein (Beyotime Biotechnology) was used to infect cells for autophagy detection. Cardiomyocytes were cultured in the adenovirus at a multiplicity of 20 of the infection for 24 h and then treated with a medium containing the appropriate reagent for 48 h in 24-well plates. Rapamycin was used as a positive control. After that, cultured cardiomyocytes were incubated with 5 *μ*g/mL DAPI for 10 min in the dark. Cells were captured under a fluorescence microscope.

### 2.7. Western Blot Analysis

Cardiomyocytes were homogenized using a lysis buffer containing 1x RIPA lysis buffer (Cell Signaling Technology, USA), 1% NaF, 1% Na_3_VO_4_, and 1% protease inhibitor cocktail. Protein concentrations were determined by a BCA protein assay (Beyotime Biotechnology). Protein was added to each lane of a 12% SDS-polyacrylamide gel, and the proteins were separated and transferred onto a PVDF membrane. The membrane was blocked and incubated overnight at 4°C with AKT, p-AKT, GSK3*β*, p-GSK3*β*, Bax, Bcl-2, Beclin-1, LC3B, Cleaved Caspase3 (1 : 1000, Cell Signaling Technology), PK2 (1 : 1000, Abcam, USA), PKR1, PKR2 (1 : 2000, Santa Cruz Biotechnology, USA), and GAPDH (1 : 5000, Proteintech, USA) antibodies. After incubation with a secondary antibody for 1 h, analysis was carried out using an ECR kit (Meilunbio). Analytical quantification was performed using Quantity One software (Bio-Rad, USA) [22].

### 2.8. Statistics Analysis

The data are presented as the mean ± SEM. All statistical analyses were performed using independent sample t-test and one-way analysis of variance. A *P* value less than 0.05 was regarded as statistically significant.

## 3. Results

### 3.1. PK2 Decreased Intracellular ROS Production in High Glucose/High Palmitic Acid-Treated Cardiomyocytes

**Figure 1:**
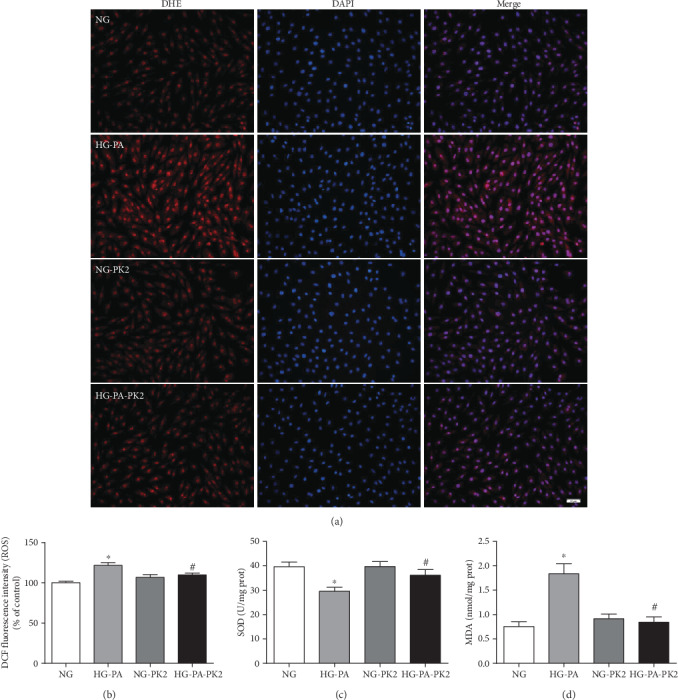
PK2 decreased intracellular ROS production in high glucose/high palmitic acid-treated cardiomyocytes. (a) Representative images of DHE staining, magnification = 200x, scale bar = 50 *μ*m, *n* = 3 independent groups. (b) Quantification of DCFH-DA staining, *n* = 8independent groups. (c) SOD level, *n* = 7‐9 independent groups. (d) MDA level, *n* = 7‐10 independent groups. NG: normal glucose; HG-PA: high glucose/high palmitic acid; NG-PK2: normal glucose plus PK2; HG-PA-PK2: high glucose/high palmitic acid plus PK2. ^∗^*P* < 0.05 versus the NG group; ^#^*P* < 0.05 versus the HG-PA group.

ROS is the key executor of oxidative stress, which causes cardiomyocyte apoptosis during DCM, so we detected ROS levels by DHE staining and DCFH-DA staining. As presented in Figures 1(a) and 1(b), the level of ROS in the high glucose/high palmitic acid group was much higher than that in the NG group, while the abnormal increase in ROS was suppressed by treatment with PK2.

SOD is the main enzyme that acts against oxygen free radical damage, and MDA is an indicator of lipid peroxidation levels. SOD activity was significantly decreased, and MDA content was significantly increased in the high glucose/high palmitic acid group. PK2 administration significantly increased SOD activity and reduced the MDA content compared with those in cardiomyocytes exposed to high glucose/high palmitic acid alone (Figures 1(c) and 1(d)).

### 3.2. Effect of PK2 on Cardiomyocyte Apoptosis and Apoptosis-Related Proteins Induced by High Glucose/High Palmitic Acid

**Figure 2:**
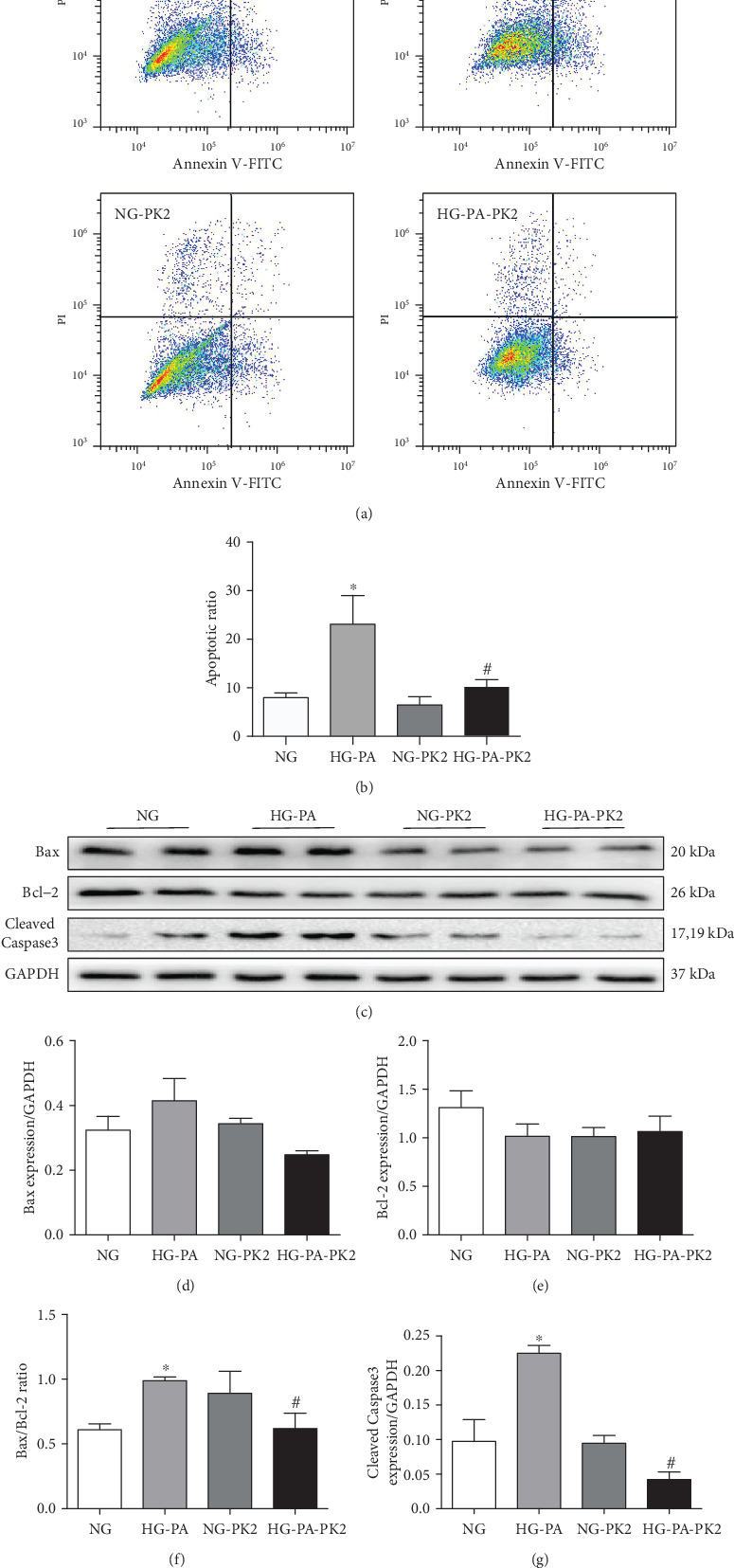
Effect of PK2 on cardiomyocyte apoptosis and apoptosis-related proteins induced by high glucose/high palmitic acid. (a) Cells were detected with a flow cytometer, *n* = 7‐11 independent groups. (b) Analysis of apoptosis. (c) Images of Bax, Bcl-2, and Cleaved Caspase3 proteins. (d) Analysis of Bax. (e) Analysis of Bcl-2. (f) Analysis of the Bax/Bcl-2 ratio. (g) Analysis of Cleaved Caspase3. ^∗^*P* < 0.05 versus the NG group; ^#^*P* < 0.05 versus the HG-PA group; *n* = 3‐4 independent groups.

To investigate the role of PK2 in cell survival, we examined apoptosis and apoptosis-related proteins by flow cytometry and western blotting. Flow cytometry showed that the number of apoptotic cardiomyocytes in the NG group was less than that in the high glucose/high palmitic acid group and that the administration of PK2 significantly abrogated the increase in apoptosis triggered by high glucose/high palmitic acid (Figures 2(a) and 2(b)).

The process of apoptosis involves changes in apoptosis-related protein expression. The data showed that the Bax/Bcl-2 ratio and Cleaved Caspase3 expression were significantly increased in the high glucose/high palmitic acid group compared to the NG group and that the Bax/Bcl-2 ratio and the Cleaved Caspase3 expression were significantly decreased after cotreatment with PK2 (Figures 2(c)–2(g)).

### 3.3. Effect of PK2 on Cardiomyocyte Autophagy-Related Proteins Induced by High Glucose/High Palmitic Acid

**Figure 3:**
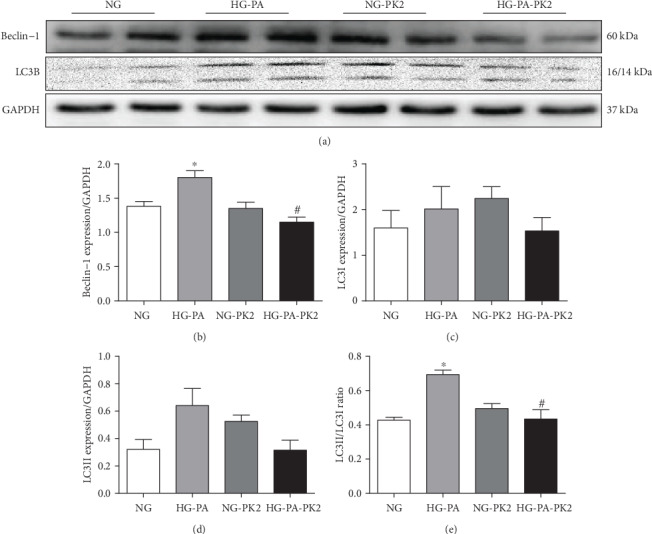
Effect of PK2 on cardiomyocyte autophagy-related proteins induced by high glucose/high palmitic acid. (a) Images of Beclin-1 and LC3B protein expression. (b) Analysis of Beclin-1. (c) Analysis of LC3I. (d) Analysis of LC3II. (e) Analysis of the LC3II/LC3I ratio. ^∗^*P* < 0.05 versus the NG group; ^#^*P* < 0.05 versus the HG-PA group, *n* = 4‐6 independent groups.

To discern the role of autophagy in the beneficial role of PK2 inactivation against high glucose/high palmitic acid toxicity, we detected autophagy-related proteins by western blotting. As shown in Figures 3(a)–3(e), the Beclin-1 expression and the LC3II/LC3I ratio were significantly upregulated in the high glucose/high palmitic acid group. However, the effect was overtly attenuated by PK2 administration.

### 3.4. PK2 Increased PKR Expression in High Glucose/High Palmitic Acid-Treated Cardiomyocytes

**Figure 4:**
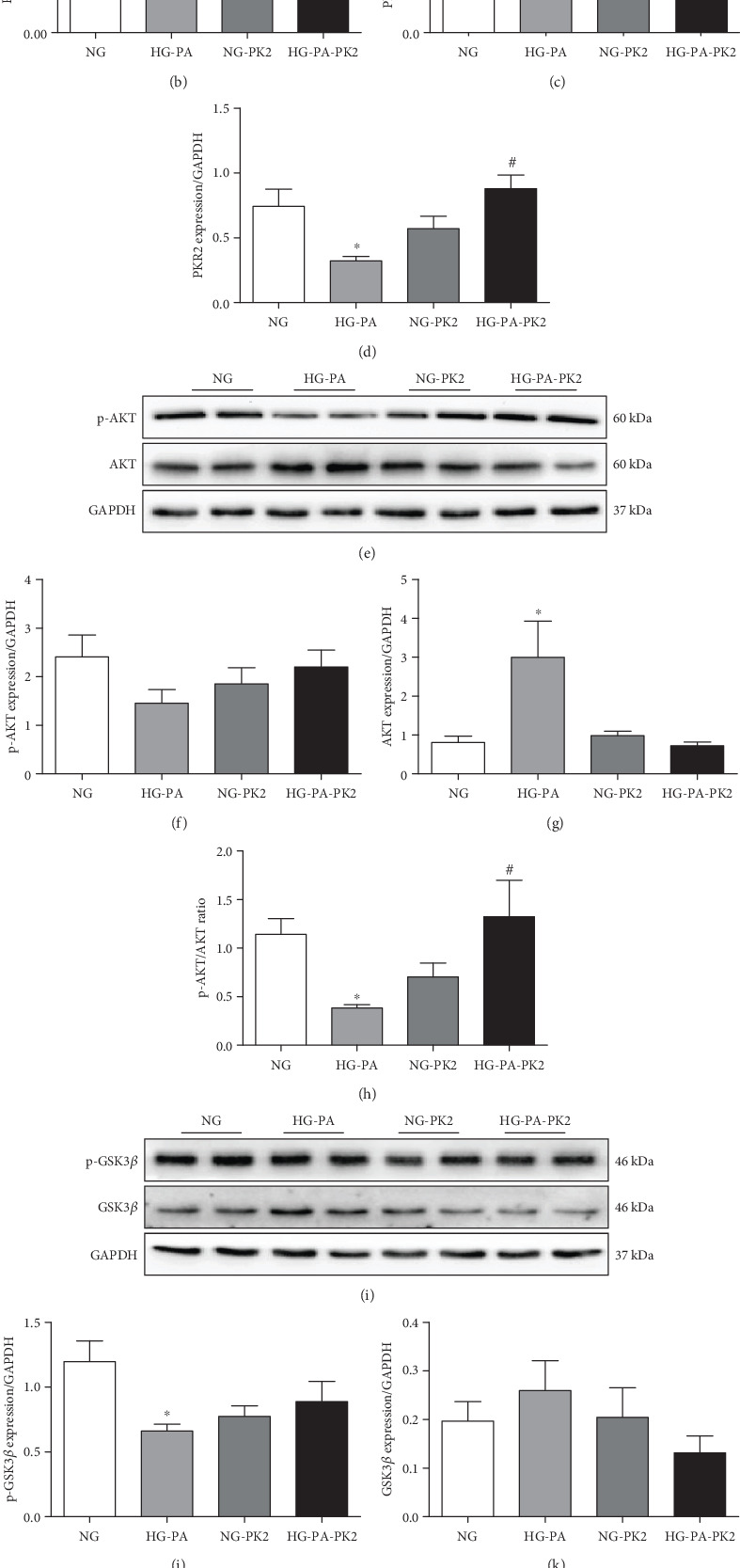
Activation of the PK2/PKR/AKT/GSK3*β* pathway by PK2 in high glucose/high palmitic acid-treated cardiomyocytes. (a) Images of PK2, PKR1, and PKR2 protein expression. (b) Analysis of PK2. (c) Analysis of PKR1. (d) Analysis of PKR2. (e) Images of p-AKT and AKT protein expression. (f) Analysis of p-AKT. (g) Analysis of AKT. (h) Analysis of the p-AKT/AKT ratio. (i) Images of p-GSK3*β* and GSK3*β* protein expression. (j) Analysis of p-GSK3*β*. (k) Analysis of GSK3*β*. (l) Analysis of the p-GSK3*β*/GSK3*β* ratio. ^∗^*P* < 0.05 versus the NG group; ^#^*P* < 0.05 versus the HG-PA group; *n* = 4‐6 independent groups.

The PK2/PKR pathway participates in cardiovascular disease [23], thus, we analysed the expression of PK2 and PKR by western blotting. As shown in Figure 4(a)-4(d), the greatly decreased PK2, PKR1, and PKR2 expression was observed in cardiomyocytes after exposure to high glucose/high palmitic acid compared to the NG group, these effects were altered in cells that received PK2 treatment. The data demonstrated that PK2 had a positive effect on high glucose/high palmitic acid-induced injury by stimulating two closely related receptors.

### 3.5. PK2 Activated the AKT/GSK3*β* Pathway in High Glucose/High Palmitic Acid-Treated Cardiomyocytes

The AKT/GSK3*β* pathway regulates cell survival, apoptosis, and angiogenesis [24], and the PK2/PKR2 pathway plays an important role by activating the crucial downstream AKT pathway [10, 19]. To study the underlying mechanisms of PK2 in high glucose/high palmitic acid-induced injury, AKT/GSK3*β* pathway proteins were detected by western blotting. The data showed that H9c2 cells displayed a significant decrease in the p-AKT/AKT and p-GSK3*β*/GSK3*β* ratios after high glucose/high palmitic acid treatment, the effect of which was reversed after cotreatment with PK2 (Figures 4(e)–4(l)), indicating that AKT/GSK3*β* signalling may be involved in the cardioprotective effect of PK2 on cardiomyocytes exposed to high glucose/high palmitic acid.

### 3.6. AKT Inhibitor Abolished the Effects of PK2 on Cardiomyocyte ROS Production and Apoptosis

To determine whether PK2 attenuated apoptosis by inhibiting oxidative stress and autophagy mediated by the AKT/GSK3*β* pathway in high glucose/high palmitic acid-damaged cardiomyocytes, the AKT inhibitor was administered to cardiomyocytes.

**Figure 5:**
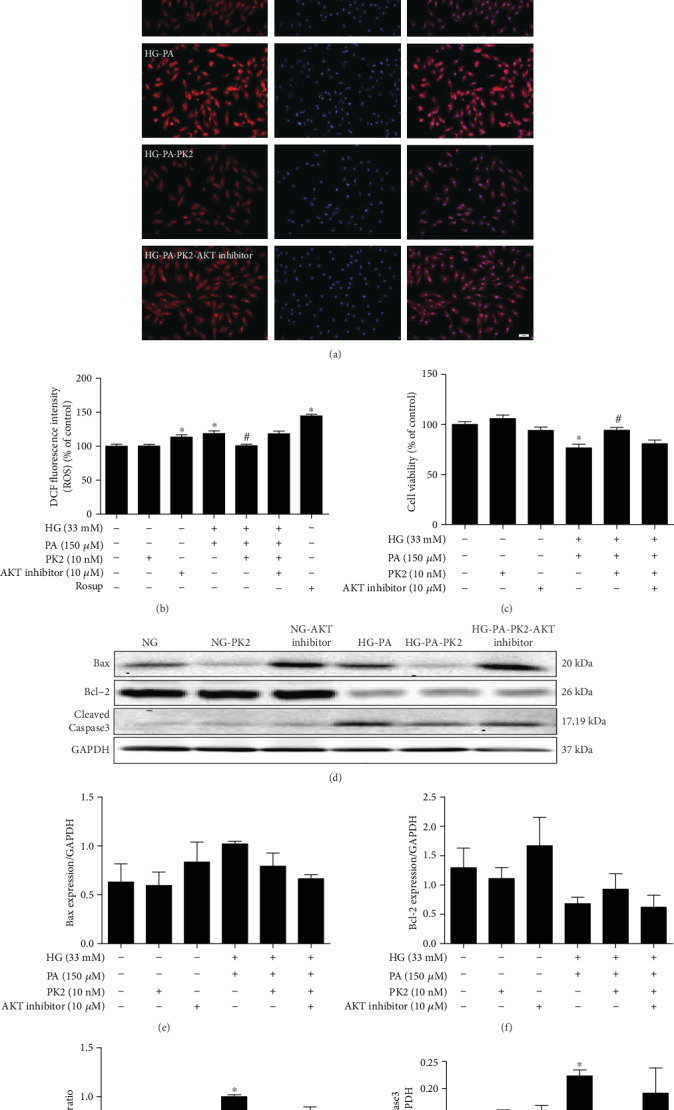
AKT inhibitor abolished the effects of PK2 on cardiomyocyte ROS production and apoptosis. (a) Representative images of DHE staining, magnification = 200x, scale bar = 50 *μ*m, *n* = 3 independent groups. (b) Quantification of DCFH-DA staining, *n* = 12‐16 independent groups. (c) Cell viability. (d) Images of Bax, Bcl-2, and Cleaved Caspase3 protein expression. (e) Analysis of Bax. (f) Analysis of Bcl-2. (g) Analysis of the Bax/Bcl-2 ratio. (h) Analysis of Cleaved Caspase3. ^∗^*P* < 0.05 versus the NG group; ^#^*P* < 0.05 versus the HG-PA group; *n* = 3‐4 independent groups. NG: normal glucose; NG-PK2: normal glucose plus PK2; NG-AKT inhibitor: normal glucose plus AKT inhibitor; HG-PA: high glucose/high palmitic acid; HG-PA-PK2: high glucose/high palmitic acid plus PK2; HG-PA-PK2-AKT inhibitor: high glucose/high palmitic acid plus PK2 plus AKT inhibitor.

As shown in Figures 5(a) and 5(b), similar to previous experimental results, PK2 decreased the accumulation of ROS, while the AKT inhibitor abolished the decrease in ROS accumulation induced by PK2.

In addition, the MTT experiment showed that high glucose/high palmitic acid decreased cell viability and that the PK2-induced increase in cell viability was blocked by the AKT inhibitor (Figure 5(c)). In parallel, the regulation of Bax/Bcl-2 ratio and Cleaved Caspase3 expression by PK2 was inhibited by AKT inhibitor treatment (Figures 5(d)–5(h)).

### 3.7. AKT Inhibitor Counteracted the Effects of PK2 on Cardiomyocyte Autophagy

**Figure 6:**
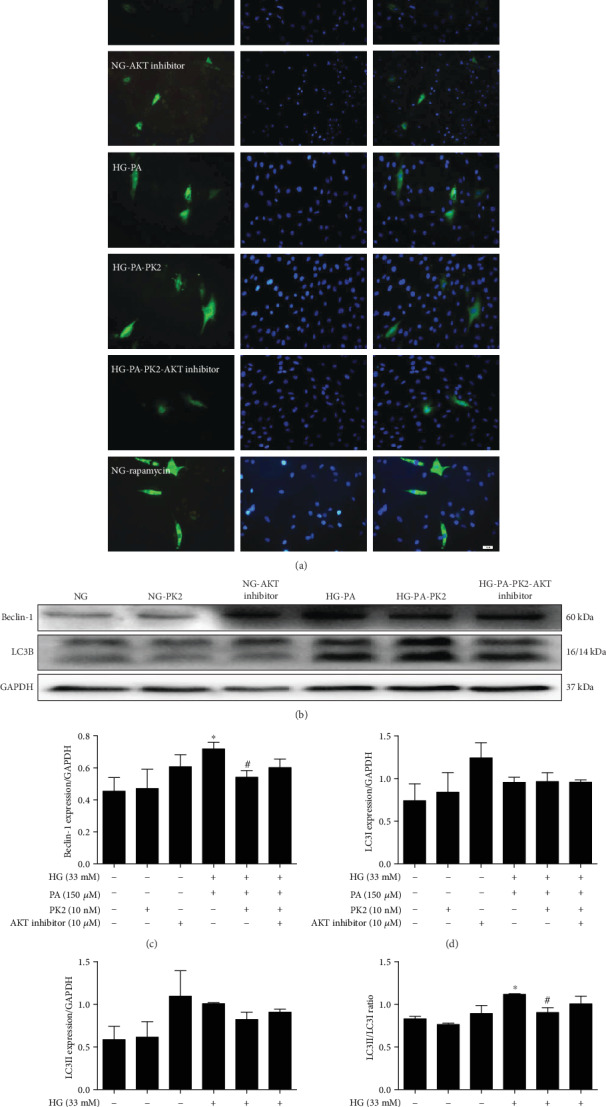
AKT inhibitor counteracted the effects of PK2 on cardiomyocyte autophagy. (a) Representative images of autophagy indicated by GFP-LC3, magnification = 200x, scale bar = 50 *μ*m, *n* = 3 independent groups. (b) Images of Beclin-1 and LC3B protein expression. (c) Analysis of Beclin-1. (d) Analysis of LC3I. (e) Analysis of LC3II. (f) Analysis of the LC3II/LC3I ratio. ^∗^*P* < 0.05 versus the NG group; ^#^*P* < 0.05 versus the HG-PA group; *n* = 3 independent groups.

To determine whether PK2 affects autophagy through the AKT pathway, the number of GFP-LC3 puncta (green fluorescence) on the autophagosome membrane and protein expression was measured. PK2 effectively rescued the increase in GFP-LC3 puncta in the high glucose/high palmitic acid group, while PK2 itself did not affect it. However, the AKT inhibitor offset the decrease in GFP-LC3 puncta induced by PK2 (Figure 6(a)). PK2 also failed to alter the expression levels of Beclin-1 and the LC3II/LC3I ratio in high glucose/high palmitic acid-treated cardiomyocytes when the AKT inhibitor was applied (Figures 6(b)–6(f)).

### 3.8. Effect of AKT Inhibitor on the PK2/PKR/AKT/GSK3*β* Pathway in Cardiomyocytes

**Figure 7:**
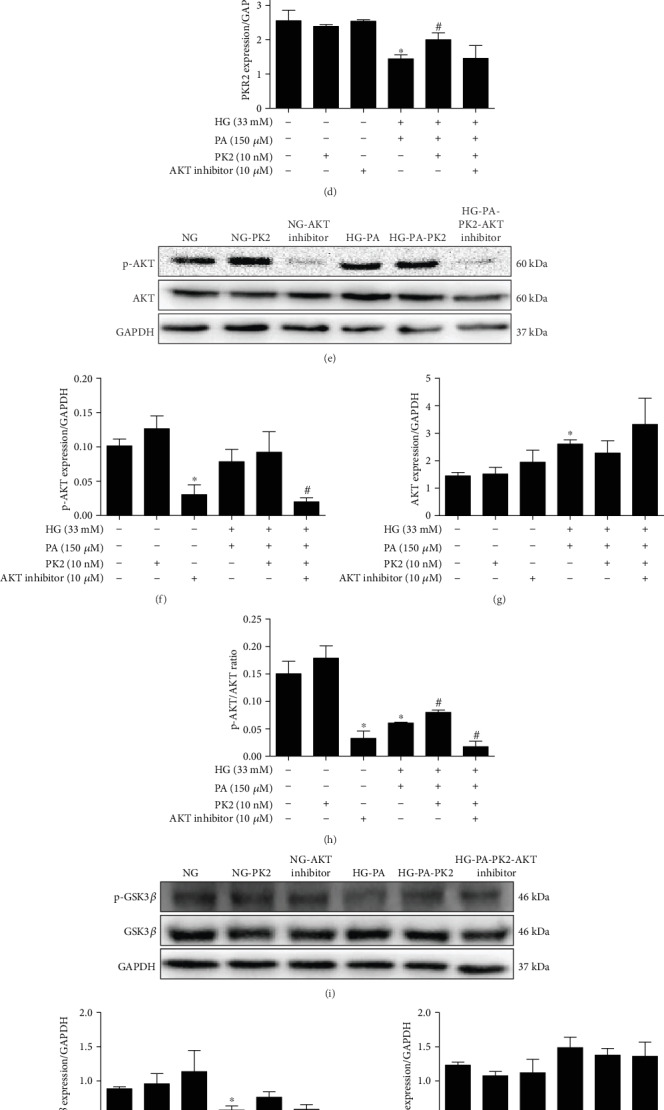
Effect of AKT inhibitor on the PK2/PKR/AKT/GSK3*β* pathway in cardiomyocytes. (a) Images of PK2, PKR1, and PKR2 protein expression. (b) Analysis of PK2. (c) Analysis of PKR1. (d) Analysis of PKR2. (e) Images of p-AKT and AKT protein expression. (f) Analysis of p-AKT. (g) Analysis of AKT. (h) The p-AKT/AKT ratio. (i) Images of p-GSK3*β* and GSK3*β* protein expression. (j) Analysis of p-GSK3*β*. (k) Analysis of GSK3*β*. (l) The p-GSK3*β*/GSK3*β* ratio. ^∗^*P* < 0.05 versus the NG group; ^#^*P* < 0.05 versus the HG-PA group; *n* = 3 independent groups.

To further explore the mechanism underlying PK2-mediated apoptosis and autophagy responses in high glucose/high palmitic acid-treated cardiomyocytes, PK2/PKR/AKT/GSK3*β* pathway proteins were detected after the addition of the AKT inhibitor. As shown in Figures 7(a)–7(d), treatment with the AKT inhibitor reversed the increased effect of PK2 and abolished the increase in PKR1 and PKR2 expression induced by PK2. Moreover, the AKT inhibitor reversed the increased effect of PK2 on p-AKT/AKT and P-GSK3*β*/GSK3*β* ratios. The data illustrated that PK2 protected cardiomyocytes from damage caused by high glucose/high palmitic acid through the activation of the AKT/GSK3*β* pathway (Figures 7(e)–7(l)).

## 4. Discussion

The findings of our current study suggest that PK2 protects against the high glucose/high palmitic acid incubation-induced impairment of ROS accumulation, apoptosis, and autophagosome accumulation by activating the PK2/PKR/AKT/GSK3*β* pathway in cardiomyocytes. The dysregulation of glucose and lipids is an important factor in the pathophysiology of DCM [25], and the clinical management of DCM remains challenging. Our research suggests that PK2 may serve as a latent therapeutic target for DCM. Our data demonstrate that PK2/PKR may play a pivotal role in myocardial damage caused by glucolipotoxicity.

Oxidative stress is a state of imbalance between oxidative and antioxidative mechanisms caused by ROS accumulation [26]. Abnormal oxidative stress levels, which exert a significant role in the pathophysiology of DCM, have pernicious effects on cellular signal transduction and induce cardiomyocyte apoptosis [27]. The disorder of glucose and lipids induces cardiac ROS accumulation and triggers apoptosis, eventually leading to cardiac remodelling in DCM [28]. Therefore, a clear reduction in ROS accumulation and cardiomyocyte apoptosis is considered an alternative strategy for protecting cardiomyocytes from glucolipotoxicity. DHE and DCFH-DA staining and oxidative stress biochemical indices indicated oxidative stress upon high glucose/high palmitic acid challenge, and these effects were mitigated by PK2. Our further examination revealed that PK2 reduced cardiomyocyte apoptosis induced by high glucose/high palmitic acid and ameliorated proapoptotic alterations including the Bax/Bcl-2 ratio and the Cleaved Caspase3 expression. Thus, it is plausible that the cardioprotective effects exerted by PK2 are mediated in part through the inhibition of ROS generation and apoptosis.

Autophagy is a conserved self-eating process that is involved in the development of multiple types of diseases, such as diabetes mellitus and cardiomyopathy [29, 30]. It is well known that appropriate autophagy maintains cardiac homeostasis, whereas elevated or defective autophagy exacerbates cardiac damage and contributes to the progression of DCM [31, 32]. Beclin-1 plays a major role in the formation of autophagosomes and lysosomal organisms by forming distinct protein complexes. Abnormal expression of Beclin-1 is deleterious for cell viability [33]. LC3B is a versatile marker protein of autophagy. LC3I in the cytosol is converted to autophagosome-bound LC3II, the content of LC3II is proportional to the number of autophagosomes, and LC3II is considered to be a marker of autophagosomes [34]. Previous reports have revealed increases in LC3II and Beclin-1 expression and the LC3II/LC3I ratio in the myocardium of mice with type 2 diabetes induced by a high-fat diet plus streptozotocin [32, 35, 36]. Therefore, reducing autophagy-related proteins is considered another strategy for protecting cardiomyocytes from glucolipotoxicity. In our study, we noted that PK2 offset the overexpression of autophagy-related proteins and autophagosome accumulation in high glucose/high palmitic acid-challenged cardiomyocytes through the following mechanisms: (1) a drastic increase in autophagosome formation induced by high glucose/high palmitic acid, which was reversed by PK2, and (2) Beclin-1 overexpression and an increase in the LC3II/LC3I ratio in H9c2 cells exposed to high glucose/high palmitic acid, which were restored by PK2.

PK2 exists in a variety of tissues, such as the brain, heart, and testes [37–39], and serves as a key factor for neuronal survival, olfactory bulb morphogenesis, and testis development [9, 39, 40]. Recently, PK2 has been found to participate in cardiac survival, proliferation, and migration [41]. PK2 has a wide array of cardiovascular effects by stimulating PKR1 and PKR2. Urayama et al. [13] found that PK2 and PKR are expressed in the cardiovascular system in both mice and humans, and the downregulation of PK2 and PKR1 has been observed in heart failure patients. Interestingly, the overexpression of PKR1 stimulates angiogenesis and protects cardiomyocytes from oxidative stress, and the loss of PKR1 induces apoptosis and ultimately impairs cardiac structure and function [13, 42]. Evidence suggests that PKR2 impairs endothelial integrity without inducing angiogenesis in cardiovascular tissues [43]. However, the precise functions of PK2 in high glucose/high palmitic acid-induced cardiomyocyte damage have not been determined. Our study showed that the expression of PK2, PKR1, and PKR2 was decreased in cardiomyocytes treated with high glucose/high palmitic acid and that the abnormal expression of PK2/PKR was effectively reversed by the administration of PK2. Therefore, we deduced that PK2 is responsible for improvements in oxidative stress and apoptosis in cardiomyocytes exposed to high glucose/high palmitic acid through stimulating PKR, but the underlying mechanism is still unknown.

AKT has been shown to be involved in cardiovascular functions linked with cell survival, growth, proliferation, and angiogenesis by inactivating its downstream target GSK3*β* [44–46]. Dariushnejad et al. [47] reported that the activation of the AKT signalling pathway in the hearts of high-fat diet- and streptozotocin-induced diabetic rats has a beneficial effect on apoptosis and angiogenesis. Furthermore, evidence has revealed that the cardioprotective actions of PK2/PKR may involve the phosphorylation of AKT, as this effect maintains oxidative stress, myocardial survival, and angiogenesis in myocardial infarction mice [13]. In addition, impaired AKT activity in response to insulin is a common feature of DCM [48]. Our study clearly shows that activating the AKT-dependent pathway may serve as a key mechanism of the cardioprotective role of PK2. This is supported by several pieces of experimental data. (1) The p-AKT/AKT and p-GSK3*β*/GSK3*β* ratios were decreased in cardiomyocytes exposed to high glucose/high palmitic acid, and the effect was reversed by PK2. Interestingly, the effect of the PK2-induced activation of the AKT pathway against high glucose/high palmitic acid was offset by the AKT inhibitor. (2) An increase in the protein level of PK2 was closely correlated with PKR1 and PKR2 expression levels induced by PK2 in cardiomyocytes, and these effects were not mediated in the presence of the AKT inhibitor. (3) The AKT inhibitor drastically increased autophagosome formation and was closely correlated with reductions in autophagy markers such as Beclin-1 and the LC3II/LC3I ratio induced by PK2. (4) The AKT inhibitor weakened the effect of PK2 on oxidative stress and apoptosis. These findings demonstrate a likely role for the AKT signalling cascade in the regulation of PK2-inhibited oxidative stress, apoptosis, and autophagosome accumulation to abnormal glucose and lipid metabolism.

In summary, the data suggest that PK2 may protect against high glucose/high palmitic acid-induced cardiomyocyte injury, including increased oxidative stress, apoptosis, and autophagosome accumulation, possibly by restoring the AKT/GSK3*β* pathway. These outcomes help to elucidate the utility of PK2 as a potential treatment target for DCM. Given the limited cardiovascular experiments performed, the clinical application of PK2 requires further scrutiny in vitro and in vivo to better define its effects in DCM.

## Data Availability

The data used to support the findings of this study are included within the article.
